# An unexpected appendiceal orifice mass

**DOI:** 10.1055/a-2849-6208

**Published:** 2026-05-05

**Authors:** Xiaojing Du, Zehua Zhang, Shuangzhu Yang, Yijun Wang, Meidong Xu, Haibin Zhang

**Affiliations:** 1 Endoscopy Center, Department of Gastroenterology 66324 Shanghai East Hospital, Tongji University School of MedicineShanghaiChina

**Fig. 1:**
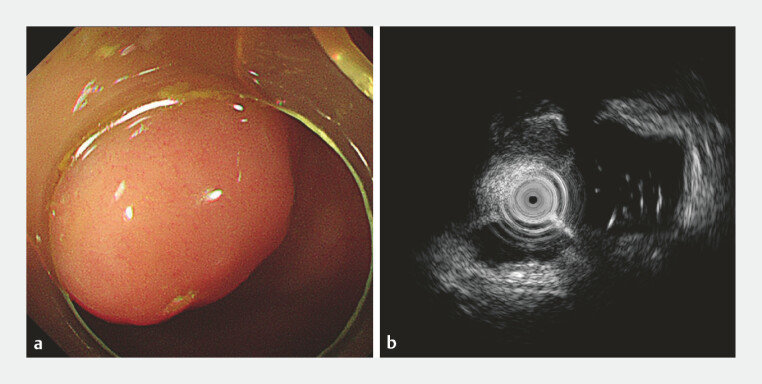
**a** Endoscopic white light manifestation. **b** Endoscopic ultrasound manifestation.

**Fig. 2:**
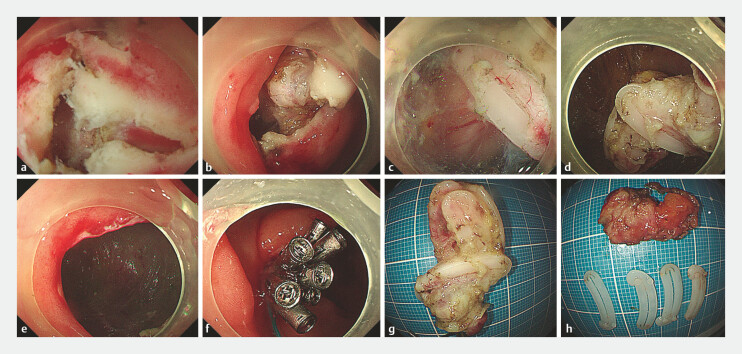
**a** Full-thickness incision. **b** Rubber band traction. **c** A white foreign body encased in granulation tissue. **d** An intraoperative view of the mass. **e** The post-resection defect. **f** The defect was closed using clips and endoloop. **g** An ex vivo specimen. **h** Four Hem-o-lok clips were separated from the mass.

A 56-year-old woman with a history of laparoscopic appendectomy (LA) 3 years prior presented with a submucosal lesion in the appendiceal orifice during colonoscopy. Computed tomography (CT) performed at an outside institution showed the absence of the appendix, with no other abnormalities. A subsequent attempt of endoscopy treatment at that institution was failed. Hence, the patient was referred to our center. Routine blood tests upon admission were normal. White-light endoscopy revealed a smooth-surfaced submucosal elevation measuring about 1.0 × 0.8 cm at the appendiceal orifice ( Fig. 1 **a** ). Endoscopic ultrasound demonstrated a hypoechoic lesion containing internal hyperechoic foci ( Fig. 1 **b** ). Subsequently, the patient underwent endoscopic full-thickness resection (EFTR; Video 1 ). After a layered full-thickness incision, we found a white foreign body encased in granulation tissue ( Fig. 2 **a–d** ). Given the potential risks associated with retained foreign bodies, the lesion was completely removed, and the defect was immediately closed with endoscopy clips and endoloop ( Fig. 2 **e** and **f** ). Upon separation of the ex vivo specimen, four white foreign bodies were identified ( Fig. 2 **g** and **h** ). Based on the appendectomy history, these were inferred to be Hem-o-lok clips. The patient was discharged on postoperative day 6 with no complications.

Removing Hem-o-lok clips by endoscopic full-thickness resection.Video 1

The Hem-o-lok clip is one of the most common techniques for appendix stump closure during LA 1 . Since Hem-o-lok clips are radiolucent, they are frequently undetectable on imaging, posing a diagnostic challenge 2 . Therefore, the iatrogenic foreign body should be strongly suspected in post-appendectomy patients presenting with appendiceal orifice mass. Fortunately, the patient was diagnosed and underwent foreign body removal via endoscopy treatment. To our knowledge, this is the first report case of using EFTR to remove post-LA Hem-o-lok clips. As a safe and efficient solution in these cases, EFTR provides definitive treatment and diagnosis while avoids the trauma of open surgery.

Endoscopy_UCTN_Code_CPL_1AJ_2AI
